# An exploratory survey on community pharmacists’ service provision for pregnant and lactating women in Sharjah, United Arab Emirates

**DOI:** 10.1371/journal.pone.0262254

**Published:** 2022-02-02

**Authors:** Zahraa Abdullatif Akkad, Muaed Alomar, Subish Palaian

**Affiliations:** 1 College of Pharmacy and Health Sciences, Ajman University, Ajman, United Arab Emirates; 2 Department of Clinical Sciences, College of Pharmacy and Health Sciences, Ajman University, Ajman, United Arab Emirates; University of South Australia, AUSTRALIA

## Abstract

Over-the-counter (OTC) medications are generally deemed safe to be used during pregnancy and lactation. However, some products can be harmful to the mother, fetus or breast-fed child, which presents a challenge to health professionals and consumers. This study was aimed at assessing the practice of OTC medication dispensing and counseling services provided to pregnant and lactating by community pharmacists (CPs). A cross-sectional descriptive questionnaire-based survey was answered during February—November 2020, by licensed CPs practicing in Sharjah, United Arab Emirates. The determination of the significant factors associated with the CPs’ views and OTC medication dispensing during pregnancy and breastfeeding was carried out using logistic regression. Among 256 respondents, dispensing medicines and referral to a physician were the predominant services provided to pregnant and lactating women. Respondents dispensed medications mostly to treat headache (74.2%), fever (62.5%) in pregnant women, and headache (81.3%) and fever (65.2%) in lactating mothers. Referral to a physician was common in pregnant women in the following cases: varicose veins (72.7%), swelling of the feet and legs (71.9%), and vaginal itching (53.9%). In breastfeeding women, the referrals were commonly for varicose veins (79.7%), swelling of the feet and legs (73.0%) and mastitis (70.3%). Most participants came to an agreement that CPs are capable of counselling and providing pregnant and lactating women the best OTC treatment. Around 35% of the respondents stated that OTC medicines are not safe to be used during pregnancy. One in five respondents stated that OTC medicines are not safe for breastfeeding women. CPs were confident to counsel and provide advice to pregnant and breastfeeding women to address medication and health problems. Proper utilization of CPs can contribute largely to the healthcare system in managing common minor ailments in pregnant and lactating women, reducing the need to visit the physician and enhancing patient safety.

## Introduction

Women may experience health problems during pregnancy and lactation that require care in order to maintain the wellbeing of mothers, fetus and their infants [1]. The physiological changes occurring during pregnancy and lactation induce ailments which are often mild and self-limiting. Prompt treatment of minor ailments is essential for the safety of the mother and the fetus. Most of health issues that mothers face during pregnancy and lactation such as headache, cough, constipation, heartburn, nausea and vomiting, and insomnia etc., can be treated with non-prescription medications and by receiving appropriate counseling from community pharmacists (CPs) [1, 2].

When individuals self-administer pharmacological substances, labeled "over- the-counter (OTC)" medications to treat common illnesses without a prescription, such behavior is known as "self-medication’’ [2, 3]. In the United States, more than 90% of women take prescription or OTC medication, [4] and globally an estimated 90% of women consume at least one medication during pregnancy [5]. OTC drugs that are commonly used during pregnancy and lactation are analgesics, cough and cold medications, laxatives, antacids, vitamins, and herbal products [6, 7].

In spite of most OTC medications having a good safety profile in pregnancy, some adverse drug reactions (ADRs) could be harmful and irreversible. In addition, the safety profile of some medications may change according to trimester [3] and hence require prudential drug selection with the help of qualified healthcare providers. As a result, in December, 2014 the US Food and Drug Administration (FDA) assigned the Pregnancy and Lactation Labelling Rule (PLLR), which provides detailed information about the safety and efficacy of medications during pregnancy and lactation [8].

The use of medications by pregnant and lactating women is concerning. Although OTC medications are generally considered safe at the usual dose, some medications can cause harm to the fetus especially in the first trimester. [3]. The use of NSAIDs during early pregnancy may increase the risk of spontaneous abortion [9]. A US FDA report published in 2020 advised against using NSAIDs from week 20. NSAIDs could cause kidney problems in the fetus which in turn lowers the amniotic fluid and increases the risk of umbilical cord compression [10]. NSAIDs should be avoided in the third trimester because of serious fetal ADRs such as: neonatal periventricular hemorrhage, renal impairment and premature closure of the ductus arteriosus (especially with indomethacin) [9, 11]. In addition, some OTC medicines may pass into breast milk, thus, posing a potential threat to the infant [12]. OTC medications should be used with caution during pregnancy and breastfeeding to avoid serious ADRs. The role of healthcare providers, including CPs, is essential when selecting the correct medications and in providing the most appropriate counselling [13, 14].

CPs are frequently the first healthcare providers who are asked for advice about medications due to their accessibility [13] which requires no prior appointment. As a result of which, pharmacists have an important role in prenatal care during pregnancy and breast-feeding which drive women to CPs for medications, counselling or both [14, 15].

Previous studies in Serbia [2], Norway [2], Kuwait [7] and Qatar [16], showed the significant role of CPs in the management of minor aliments during pregnancy. In Kuwait for instance, findings showed that pregnant and breastfeeding women frequently CPs regarding minor health problems [7].

A previous study from the UAE reported that 40% of pregnant women took OTC medications during pregnancy [17]. However, there are no studies conducted in the UAE to highlight the services offered by CPs to women during pregnancy or breast-feeding. It is important to understand what services are currently offered by CPs in order to offer recommendations for possible improvements to both services and policies. Therefore, an investigation of CPs’ behaviors and identifying services offered during pregnancy and breastfeeding needs to be explored. The purpose of this research was to assess the practice of community pharmacists, regarding counseling services and OTC medication dispensing to lactating and pregnant women. It aimed to evaluate CPs’ background knowledge and confidence in providing counselling services to pregnant and lactating women, and to identify the set of key demographic and baseline factors that are significantly associated with OTC medication dispensing in pregnancy and lactation.

## Methods

### Study design

This is a descriptive cross-sectional survey-based study conducted using a convenience sample of licensed CPs located in Sharjah, United Arab Emirates.

### Study settings

Sharjah is one of the seven Emirates in the United Arab Emirates. There are around 1300 pharmacists in Sharjah, which has the second largest number of licensed pharmacists in the United Arab Emirates after Abu Dhabi [18].

### Ethical approval

Ethical approval for performing this study was obtained from the Ethical Research Committee at Ajman University [Approval letter No: P-F-H-2020-02-27]. Written consent was obtained from all respondents prior to their participation in the research. There were no minors (under the age 18 years) involved in the research. All the ethical principles laid out by the approving body have been followed strictly.

### Sampling method & sample selection

The sample size was calculated by using the formula $n=\frac{z^{2}P(1-P)}{d^{2}}$, P = 0.5 (50%), z = 1.96, d = 0.05 (5%) [19]. These 256 licensed pharmacists were further selected from CPs in Sharjah using the convenience sampling method similar to other studies [2, 7]. Data collection was conducted within the period of February 2020- November 2020 in the midst of the COVID-19 pandemic.

### Method of data collection

A self-administrated, paper-based questionnaire was used in the beginning of data collection and subsequently the data collection was switched to online mode due to theCOVID-19 pandemic lockdown.

### Inclusion criteria

Participants were chosen randomly from both genders, aged 21 years old or above and from different nationalities. Trainee pharmacists were excluded from the study. All licensed pharmacists working in CPs, including from chain pharmacies, from all shifts were involved in data collection.

### Study tool

In this study, a questionnaire was used to answer the research questions. This questionnaire has been adopted from a previous study done in Kuwait after taking permission, and with minor modifications done to suit the present research [7]. The questionnaire had four sections (A, B, C, D). Section A was about demographic information, it is composed of 7 questions. Section B had29 questions that assessed the CP’s knowledge of OTC medication counseling during pregnancy and breastfeeding. Section C had 6 questions related to services provided to women during pregnancy and lactation. Section D had 11 questions about pharmacist’s views about self-care in pregnancy and lactation.

### Questionnaire coding

The OTC medication dispensing during pregnancy was measured by 13 questions (Q8-Q20) with categorical responses: (yes/no). If the respondent selects “yes”, the given score was 1 point, while “No” was scored 0 points. Then, the summation of scores was calculated and ranged from a minimum of “0″ to a maximum of “13″ for each participant. Similarly, OTC medication dispensing during breastfeeding was measured by 16 questions (Q 21-Q36) with categorical responses: (yes/no). If the respondent selected “yes”, the given score was 1 point, while “No” was scored 0 points. Then, the scores summation was calculated and ranged from a minimum of “0″ to a maximum of “16″ for each participant. Views about pregnancy and lactation’s self-care was measured by 10 questions with categorical responses: (agree/disagree/neutral). A ‘positive view’ answer was given a score of “1 point”, while a “negative view” answer was scored “0 points”. Then, the scores summation was calculated and ranged from a minimum of “0″ to a maximum of “10″ for each participant. A total score of ≥6 was defined as having a positive view while a score of < 6 was defined as having a negative view.

### Statistical analysis

SPSS version 26 was used in analyzing the collected data. Variables were summarized using frequencies and percentages. The determination of the significant predictors associated with the CPs’ view and OTC medication dispensing during pregnancy and breastfeeding was done by using multinomial logistic regression. The predictor variables such as gender, age, marital status, educational level and years of experience were considered covariates in the regression model. All covariates which were significant in the univariate model were considered by following the principle of parsimony and thus adjusting the effect of confounders. A p value < 0.05 was considered statistically significant at 95% CI.

## Results

### Demographic characteristics of the study participants

A total number of 256 participated in the study. Among these participants, 47.7% (n = 122) were males and 52.3% (n = 134) were females. Of the total participants, 154 (60.2%) were aged 21–30 years, 84 (32.8%) were aged 31–40 years and 18 (7%) were aged > 40 years. Among the participants, 46.9% (n = 120) were single and 53.1% (n = 136) were married. The participants were predominantly holding bachelor certificates (n = 211, or 82.4%). Among the participants, 42.6% (n = 109) graduated from local universities and 57.4% (n = 147) graduated from regional universities. Among the total participants, 119 reported having had less than 5 years of professional experience (46.5%) and 137 participants reported having ≥5 years of professional experience.

### Evaluation of community pharmacists’ knowledge regarding OTC medications counseling approaches to pregnant and breastfeeding women

Findings regarding the CPs’ counseling and OTC medicine dispensing during pregnancy shown that headache (74.2%), fever (62.5%) and gastroesophageal reflux disease (GERD)/indigestion (57.8%) were the common symptoms the CPs would recommend OTC medications for during pregnancy, see Table 1.

**Table 1:** Number and percentage of the questions on CPs knowledge about OTC medications and counseling during pregnancy (n = 256).

Counseling cases	Refer to a doctor		Dispense medicine		Provide only advice without dispensing medicine		Recommending vitamins and food supplements	
	F	%	F	%	F	%	F	%
**Headache**	12	4.7	190	74.2	45	17.6	9	3.5
**Cough, runny nose, sore throat**	59	23.0	91	35.5	69	27.0	37	14.5
**Constipation**	40	15.6	138	53.9	47	18.4	31	12.1
**Nausea/vomiting**	79	30.9	137	53.5	11	4.3	29	11.3
**GERD and indigestion**	67	26.2	148	57.8	29	11.3	12	4.7
**Diarrhea**	136	53.1	80	31.3	27	10.5	13	5.1
**Hemorrhoids**	130	50.8	48	18.8	46	18.0	32	12.5
**Insomnia**	124	48.4	3	1.2	87	34.0	42	16.4
**Varicose vein**	186	72.7	8	3.1	61	23.8	1	0.4
**Swelling of the feet and legs**	184	71.9	3	1.2	64	25.0	5	2.0
**Vaginal itching**	138	53.9	79	30.9	36	14.1	3	1.2
**Back pain**	76	29.7	105	41.0	67	26.2	8	3.1
**Fever**	70	27.3	160	62.5	26	10.2	0	0

Abbreviations: F; frequency, %; percentages, GERD; gastroesophageal reflux disease.

While the results regarding CPs’ counseling and OTC medication dispensing during the breastfeeding show that headache (81.3%) and fever (65.2%) were the most common symptoms the CPs had dispensed OTC medications for during breastfeeding, see Table 2.

**Table 2:** Number and percentage of the questions on knowledge regarding OTC medications counseling during breast-feeding (n = 256).

Counseling cases	Refer to a doctor		Dispense medicine		Provide only advice without dispensing medicine		Recommending vitamins and food supplements	
	F	%	F	%	F	%	F	%
**Mastitis**	180	70.3	41	16.0	29	11.3	6	2.3
**Sore or cracked nipple**	23	9.0	154	60.2	31	12.1	48	18.8
**Insufficient milk ejection**	81	31.6	76	29.7	36	14.1	63	24.6
**Headache**	20	7.8	208	81.3	23	9.0	5	2.0
**Cough, runny nose, sore throat**	78	30.5	129	50.4	34	13.3	15	5.9
**Constipation**	44	17.2	152	59.4	53	20.7	7	2.7
**Nausea/vomiting**	80	31.3	132	51.6	36	14.1	8	3.1
**GERD and indigestion**	56	21.9	143	55.9	56	21.9	1	0.4
**Diarrhea**	111	43.4	114	44.5	18	7.0	13	5.1
**Hemorrhoids**	137	53.5	71	27.7	25	9.8	23	9.0
**Insomnia**	133	52.0	23	9.0	50	19.5	50	19.5
**Varicose vein**	204	79.7	7	2.7	43	16.8	2	0.8
**Swelling of the feet and legs**	187	73.0	24	9.4	40	15.6	5	2.0
**Vaginal itching**	128	50.0	105	41.0	23	9.0	0	0
**Back pain**	92	35.9	122	47.7	33	12.9	9	3.5
**Fever**	81	31.6	167	65.2	5	2.0	3	1.2

Abbreviations: F; frequency, %; percentages, GERD; gastroesophageal reflux disease.

### Evaluation of services offered by the community pharmacists towards self-care during pregnancy and lactation

Of the respondents, 186 (72.7%) [95%CI: 67.2–78.1] of the participants claimed to have experience in offering counselling services to pregnant women. Among these 186 participants, 50 (32.3%) had provided the pharmaceutical care services for an average one pregnant woman weekly. The symptoms that pregnant women most frequently consulted the pharmacist for amongst the 186 participants were nausea/vomiting (43%) and headache/ back pain/ fever (31.7%) (Table 3).

**Table 3:** Number and percentage of the questions on the services provided regarding self-care by the community pharmacists in pregnancy and lactation (n = 256).

Services provided	Responses	Frequency	Percentage
**Experience in providing services for pregnant women**	Yes	186	72.7%
	No	70	27.3%
**Number of pregnant women receive your services in this pharmacy per week** **(n = 186)**	1	60	32.3%
	2–3	77	41.4%
	≥4	49	26.3%
**The symptom that pregnant women most frequently consulted** **(n = 186)**	Headache/ back pain/ fever	59	31.7%
	Constipation/diarrhea	9	4.8%
	Cough/ runny nose/sore throat	20	10.8%
	Nausea/vomiting	80	43%
	Indigestion/ GERD	7	3.8%
	Hemorrhoids	9	4.8%
	Other	2	1.1%
**Experience in providing services for breastfeeding women**	Yes	210	82%
	No	46	18%
**Number of breastfeeding women receive your services in this pharmacy per week** **(n = 210)**	1	76	36.2%
	2–3	90	42.9%
	≥4	44	21%
**The symptom that breastfeeding women most frequently consulted** **(n = 210)**	Headache/ back pain/ fever	36	17.1%
	Constipation/diarrhea	7	3.3%
	Cough/ runny nose/sore throat	53	25.2%
	Nausea/vomiting	11	5.2%
	Indigestion/ GERD	13	6.2%
	Insufficient milk	42	20%
	Sore or cracked nipple	48	22.9%

In the current study, 210 (82%) [95%CI: 77.3–86.8] of the participants claimed to have experience in offering services to breastfeeding women. Among these 210 participants, 76 (36.2%) had provided pharmaceutical care services for an average one breastfeeding woman on a weekly basis. The symptoms that breastfeeding women most frequently consulted the pharmacist for amongst the 210 participants were cough/ runny nose/sore throat (25.2%) **Table 3**.

### Evaluation of the community pharmacists’ views about self-care in pregnancy and lactation

**Table 4** presents the results of each question related to CPs’ knowledge and confidence, undergraduate pharmacy training and the safety of OTC medications in pregnancy and lactation. Websites (56.6%) were the most common sources of information the CP used regarding the symptoms during pregnancy and breastfeeding. Better views about self-care in pregnancy and lactation were observed in postgraduates (AOR 3.81; 95% CI 1.79–8.09) and those who graduated from regional universities (AOR 2.02; 95% CI 1.22–3.34).

**Table 4:** Number and percentage of the questions regarding pharmacists’ views about self-care in pregnancy and lactation.

*Views items*	*Agree*		*Neither disagree or agree*		*Disagree*	
	*F*	*%*	*F*	*%*	*F*	*%*
***OTC medication Safety***						
**OTC medicines are safe for pregnancy**	*38*	*18*.*1*	*96*	*45*.*7*	*76*	*36*.*2*
**OTC medicines are safe for breastfeeding**	*74*	*35*.*2*	*93*	*44*.*3*	*43*	*20*.*5*
**Knowledge and Confidence about Pregnancy and Lactation**						
**Community pharmacists are qualified to provide advice and an over- the-counter (OTC) therapy to treat common and minor symptoms in pregnant women**	*154*	*73*.*3*	*49*	*23*.*3*	*7*	*3*.*3*
**Community pharmacists are qualified to provide advice and an OTC therapy to treat common and minor symptoms in breastfeeding women**	*158*	*75*.*2*	*42*	*20*.*0*	*10*	*4*.*8*
**I am confident about giving advice and counseling to breastfeeding women**	*165*	*78*.*6*	*39*	*18*.*6*	*6*	*2*.*9*
**I have sufficient knowledge to solve medication and health problems of breastfeeding women**	*125*	*59*.*5*	*70*	*33*.*3*	*15*	*7*.*1*
***Undergraduate Pharmacy Training***						
**Pharmacy school provided appropriate training regarding advice and OTC therapy for pregnant women**	*84*	*40*.*0*	*85*	*40*.*5*	*41*	*19*.*5*
**Pharmacy school provided appropriate training regarding advice and OTC therapy for breastfeeding women**	*96*	*45*.*7*	*69*	*32*.*9*	*45*	*21*.*4*

Abbreviations: F; frequency, %; percentages.

### Factors influencing the OTC medication dispensing during the pregnancy and breastfeeding

In the present study, among the respondents who stated that they provided pharmaceutical care services to pregnant or breastfeeding women, significantly increased OTC medication dispensing throughout the pregnancy period was associated with male respondents (AOR 1.187; 95% CI 1.002–1.406) and in the participants with less than 5 years’ experience (AOR 1.280; 95% CI 1.046–1.566). On the other hand, decreased OTC medication dispensing was significantly associated with participants aged 21–30 years (AOR 0.569; 95% CI 0.417–0.778), participants aged 31–40 years (AOR 0.409; 95% CI 0.304–0.551) and those who graduated from local universities (AOR 0.627; 95% CI 0.508–0.774). **(Table 5).**

**Table 5:** Multinomial logistic regression analysis for the predictors associated with the OTC medications dispensing during pregnancy.

Factors	Over-The-Counter medications dispensing by community pharmacists to pregnant women			
	OTC medications dispensing score			
	AOR	95% CI		P-value
**Gender (Ref. female)**				
**Male**	1.187	1.002	1.406	0.048
**Age (Ref. > 40 years)**				
**21–30 years**	0.569	0.417	0.778	< 0.001
**31–40 years**	0.409	0.304	0.551	< 0.001
**Marital status (Ref. Married)**				
**Single**	0.846	0.686	1.042	0.115
**Educational level (Ref. Bachelor of Pharmacy)**				
**Post graduate**	0.838	0.669	1.049	0.838
**University of graduation (Ref. Outside the country)**				
**United Arab Emirates**	0.627	0.508	0.774	< 0.001
**Experience years (Ref. ≥5 years)**				
**<5 years**	1.280	1.046	1.566	0.016

P-values less than 0.05 were considered statistically significant, **Abbreviations**: AOR, adjusted odds ratio; CI, confidence interval.

Significantly increased OTC medication dispensing during breastfeeding, was observed in participants aged 21–30 years (AOR 2.037; 95% CI 1.532–2.709). However, decreased OTC medication dispensing during breastfeeding, was observed in male respondents (AOR 0.790; 95% CI 0.681–0.917) and those who graduated from local universities (AOR 0.429; 95% CI 0.357–0.515). **(Table 6).**

**Table 6:** Multinomial logistic regression analysis for the predictors associated with the OTC medications dispensing during breastfeeding.

Factors	OTC medications dispensing during breastfeeding			
	OTC medications dispensing score			
	AOR	95% CI		P-value
**Gender (Ref. female)**				
**Male**	0.790	0.681	0.917	0.002
**Age (Ref. > 40 years)**				
**21–30 years**	2.037	1.532	2.709	< 0.001
**31–40 years**	1.526	1.165	1.998	0.002
**Marital status (Ref. Married)**				
**Single**	0.927	0.775	1.109	0.408
**Educational level (Ref. Bachelor of Pharmacy)**				
**Post graduate**	0.972	0.802	1.178	0.775
**University of graduation (Ref. Outside the country)**				
**United Arab Emirates**	0.429	0.357	0.515	< 0.001
**Experience** years (Ref. ≥5 years)				
**<5 years**	0.880	0.739	1.047	0.149

P-values less than 0.05 were considered statistically significant, **Abbreviations**: AOR, adjusted odds ratio; CI, confidence interval.

## Discussion

This research evaluated the services offered by CPs in managing minor ailments during pregnancy and breastfeeding. The majority of respondents confirmed that they have experience in offering counseling services to pregnant and breastfeeding women. As far as the authors know, this study is the first of its kind in the United Arab Emirates.

### Evaluation of community pharmacists’ perspectives on practices regarding OTC medications and counseling approaches during pregnancy and breastfeeding

In the present study, the respondents were uncertain when it came to dispensing medications or referral to a physician. They focused less on non-pharmacological treatments and rarely dispensed vitamins or dietary supplements. This finding was in line with studies conducted in Kuwait and Thailand [7, 20]. The findings suggest for the need for educational interventions aimed at improving pharmacists knowledge on non-drug therapy related issues. In the current study, more than half of the respondents referred pregnant and breastfeeding women to physicians for the following cases: varicose veins, swelling of the feet and legs, and vaginal itching. Patients were referred to physicians, as CPs probably considered these symptoms might represent a more serious underlying problem. Furthermore, pregnant and breast-feeding women are considered a special population for whom even a minor drug therapy error can be harmful. In addition, it is also worth mentioning that most CPs in the UAE do not have adequate private areas within the pharmacy for history taking, physical examination and counseling and hence provide a limited scope for pharmacists to handle these patients. Improving the physical infrastructure of pharmacies and providing physical assessment skills training can be helpful in overcoming this issue.

It has been found that CPs prefer medication dispensing to treat common symptoms such as: headache (74%), fever (63%) followed by gastroesophageal reflux disorder/indigestion (58%) during the pregnancy. Medication dispensing for the treatment of the most common symptom (headache) seems consistent with previous studies conducted in Kuwait, Thailand and France (80%), (82%), (94%), respectively [7, 20, 21]. In an Ethiopian study, 61.8% of the respondents involving both hospital and CPs knew paracetamol is safe during pregnancy [22]. These observations could be due to the fact that headache can be simply treated with analgesics that are available as OTC such as paracetamol [23]. Women may suffer from headaches during pregnancy due to hormonal changes and it may improve during pregnancy. However, headaches might be a symptom of an underlying disease such as: meningitis or hypertension, which need referral to a physician for further evaluation [24].

In this study, headache (81%), fever (65%), and sore or cracked nipples (60%), were the most common symptoms the CPs had dispensed OTC medications for during breastfeeding. These results are similar to the studies in Kuwait and Thailand in dispensing medicines mostly for treating headache (83%) and fever (84%), respectively [7, 20]. Community pharmacists should be aware of the fact that headache and fever might be a symptom of undiagnosed diseases, thus, evaluation of the patient case by CP is to give the appropriate counselling and to limit excess OTC medications dispensing [24].

### Evaluation of services provided by the community pharmacists to pregnant and lactating women

In the present research, 73% of the respondents claimed that they have experience in offering services to pregnant women and 82% to breastfeeding women. Comparing these results to Thailand study, it has been found that a lower percentage of pharmacists have experience in offering services to pregnant (45%) and breastfeeding women (42%) [20]. The current results revealed that pregnant women most often consulted experienced CPs about the symptoms: nausea/vomiting, headache/ back pain/ fever and cough/ runny nose/sore throat. In addition, pharmacists were frequently consulted by breastfeeding women regarding cough/ runny nose/sore throat, sore or cracked nipples and insufficient milk. Kuwaiti CPs consulted pregnant women mostly about gastrointestinal tract symptoms (nausea & vomiting, constipation and stomach cramps), symptoms related to respiratory tract system (common cold and cough) and back pain. While lactating women asked pharmacists for a consultation regarding increasing the milk production (insufficient milk), and respiratory related symptoms (common cold and cough) [7]. In Thailand, CPs were consulted mostly about the common cold by pregnant and breastfeeding women [20]. These findings throw light on the fact that the dispensing of OTC governmental regulations in these countries are similar.

Respondents were asked about their opinions about OTC safety in pregnancy and lactation, and their confidence and knowledge to give advice and counselling service and to solve medications and health problems related to pregnant and lactating women. The majority of the participants in this study agreed that CPs are capable of giving advice and OTC therapy to treat minor ailments in pregnant and breastfeeding women. This result seems consistent with previous studies done in Kuwait and Belgium [7, 25].

More than one quarter of the respondents stated that OTC medicines are not safe to be used during pregnancy. All previous studies in different countries (Kuwait, Thailand, and Ethiopia) revealed a consistent result with this study regarding the safety of the OTC medicines during pregnancy [7, 20, 22].

Likewise, one fifth of respondents disagreed about the safety of OTC therapy in lactation. This finding was in line with Kuwait and Thailand’s studies, but a higher number of participants in these studies considered OTC medications not safe in breastfeeding compared to this study [7, 20]. Greater awareness regarding the safety and the risk that is associated with the misuse of OTC medications is essential to CPs.

### Evaluation of the community pharmacists’ background knowledge and confidence to provide counselling services to pregnant and lactating women

In the present study, respondents were confident to counsel and provide advice to pregnant and breastfeeding women to address medication and health problems (82%,79%) and they had sufficient knowledge (72%,60%), respectively. Comparing these results to previous studies, Ethiopian CPs got the highest level of confidence when providing counselling services to pregnant women (92%), whilst Qatari CPs scored the lowest level of confidence (33.3%) [22, 26]. Despite Ethiopian pharmacists being highly confident, their knowledge to counsel pregnant women was variable (48%) [22]. Level of knowledge of respondents in this study was in line with Kuwaiti study, however, respondents were more confident to offer counselling services to pregnant and lactating women [7]. Findings in this study showed that pharmacy schools did not provide appropriate training regarding OTC therapy during pregnancy and lactation. This result is related closely to the finding of a previous study done in Kuwait [7]. These findings can be explained, as Gulf countries provide similar curriculum and undergraduate training programs and required further improvement. It is also recommended that the pharmacy curricula in the region focus adequately on CPs as it has a huge scope to evolve and employ many future pharmacists.

In addition, the current study and the Kuwaiti study were similar in the most commonly used sources of information used to search about medicine use during pregnancy and breastfeeding; websites and books followed by medical journal articles [7]. Contrary to Thailand’s study, where books were the most common source of information followed by medical journal articles and websites [20]. Based on the present research finding, CPs prefer to use websites as the most common source of information due to easier accessibility. Nonetheless, it should not be always used as it can be considered a not trusted source of information [27]. In addition, CPs should be taught critical appraisal skills while interpreting medical literature. This can be provided through continuing pharmacy education programs and other educational initiatives.

### Factors influencing the OTC medication dispensing during the pregnancy and breastfeeding

This research showed a significant association between OTC medication dispensing during pregnancy with male respondents and in the participants with less than 5 years’ experience. Pharmacists with only a few years of experience (less than 5 years) would dispense medicines according to their customer’s desire, as a result, customers were satisfied by getting what they asked for. This may lead to irrational use of medicines, especially if the pharmacists have insufficient experience and medical knowledge background. In addition, most newly employed, inexperienced pharmacists would avoid losing their clients if they refused to sell medicines, so they will achieve the financial targets that pharmacy management force their employees to achieve to avoid being fired.

It was found that increased OTC medication dispensing during breastfeeding was observed among male pharmacists in spite of the claim that women may feel more comfortable discussing their lactation-related problems with female pharmacists. OTC medications dispensing was significantly decreased with participants aged 21–40 years, and those who graduated from local universities. However, better views were observed in postgraduates and those participants who graduated from regional universities. It can be concluded from the findings that undergraduate pharmacy curricula in the local universities should be improved and more training courses should be provided to undergraduates and CPs in maternal-fetal area.

### Strengths and limitations

This study is the first of its kind in the United Arab Emirates. In addition, this study highlighted a very important topic and provided valuable information worth the attention of pharmacists and researchers. However, this research is subjected to various limitations. Firstly, the current study was conducted during the COVID-19 pandemic; hence, the primary limitation to the generalization of these results is the sample size. The sample size was supposed to be 385 (target sample size), while in this study the sample size was only 256, which is considered a limitation. There were difficulties in data collection, which is considered a main barrier to collect an adequate number of samples. Secondly, the study was carried out in Sharjah Emirate only out of a possible seven Emirates. Hence the study’s findings may not represent the views and practice in other Emirates in the country. Third, Dunning-Kruger effect can occur because it is a self-administered questionnaire, in addition, it is a quantitative descriptive study which would not explain the possible reasons of inadequate knowledge. Fourth, the cut-off score for positive and negative views were fixed at the middle point (≥6 considered positive and < 6 as negative; the total score being 10). This mathematical segregation may not actually represent the real views of the respondents and hence can be a potential limitation.

## Conclusions

The CPs are confident to counsel and provide advice to pregnant and breastfeeding women to address medication and health problems and they have sufficient knowledge. Few pharmacists clearly indicated that OTC medicines are safe during pregnancy and breastfeeding. Regarding self-care in pregnancy and lactation, postgraduates and those participants who graduated from regional universities had positive views. Pharmacy schools and pharmaceutical associations in the UAE should design new strategies to improve the CP’s role as a healthcare provider with continuous professional development programs. Proper utilization of CPs can save physicians’ time, provides convenience to pregnant and lactating mothers and enhances patient care in managing minor ailments.

## Recommendations

According to the outcomes determined from this study, the following recommendations can be made:

- Encouraging more pharmacists to participate in such research from other Emirates to have more accurate results (All seven Emirates should participate to have more representative data).
- Encouraging pharmacy schools and pharmaceutical associations in UAE to design new strategies to improve the CP’s role as a healthcare provider with continued professional training.
- Urgent improvements are needed on the undergraduate pharmacy curricula in the local universities, to fill the knowledge gaps of maternal-fetal medicine.

## Supporting information

S1 File Questionnaire form. (PDF)

S1 Data Research data. (SAV)
