# Supplementary material for: An exploratory survey on community pharmacists’ service provision for pregnant and lactating women in Sharjah, United Arab Emirates
Source: PLoS One. 2022 Feb 2;17(2):e0262254. doi: 10.1371/journal.pone.0262254 (PMC8809596; doi:10.1371/journal.pone.0262254)
Supplement: S1 File — (PDF) [file pone.0262254.s001.pdf]

College of Pharmacy & Health Sciences

Consent Form

Dear Participant,<br><br>I'm a master pharmacy student at Ajman University.<br><br>This study aimed to evaluate the services provided by community pharmacist to pregnant and lactating women.<br><br>I would be very grateful if you can help me with this study by completing this questionnaire.<br><br>This survey is confidential.-<br><br>- Participation in this research is completely voluntary and you may refuse to participate without any You’ll receive No compensation for & consequences participating in this survey.<br><br>- The questionnaire will take few minutes to complete **(Less than 10 minutes).**<br><br>.	عزيزي المشارك،<br><br>أنا طالبة ماجستير من كلية الصيدلة في جامعة عجمان.<br><br>تهدف هذه الدراسة إلى تقييم الخدمات الطبية التي يقدمها صيدلي المجتمع إلى النساء الحوامل و المرضعات.<br><br>سأكون ممتنة جدا اذا كنت تستطيع مساعدتي في هذه الدراسة من خلال استكمال هذا الاستبيان.<br><br>- هذا الإستبيان يحافظ على سرية المعلومات المعطاة.<br><br>- المشاركة في هذا الإستبيان أمر اختياري و بإمكانك رفض المشاركة إذا لم ترغب في ذلك و لن يتلقى المشارك في الإستبيان أية حوافز مادية أو غيرها.<br><br>- هذا الإستبيان لن يأخذ سوى بضع دقائق من وقتك<br><br>لإستكماله **(أقل من 10 دقائق).**

Thank you for your Co-operation

شكرا لتعاونكم و مشاركتكم في هذا الإستبيان

A. Some Questions about you.

1) Gender? (الجنس)
- Female/ أنثى
- Male/ ذكر

2) Age? (العمر؟)
- 21-30
- 31-40
- 40<

3) Your marital status? (ما هي حالتك الإجتماعية؟)
- Singleأعزب\عزباء
- Married متزوج\متزوجة

4) What is your qualification in pharmacy? (ما هي مؤهلاتك الدراسية؟)
- Bachelor of Pharmacy بكالوريوس في الصيدلة
- Master of Pharmacy ماجستير في الصيدلة
- Doctor of Pharmacy (Pharm D) دكتور في الصيدلة

5) Country of Graduation from Pharmacy school? (الدولة التي حصلت فيها على مؤهلك الدراسي؟)
- United Arab Emirates دولة الإمارات العربية المتحدة
- Outside the country خارج دولة الإمارات العربية المتحدة

6) Your experience as practitioners in years? (خبرتك في مهنة الصيدلة بالسنوات؟)
- <5
- ≥5

7) You're Pharmacy Location? (موقع الصيدلية التي تعمل بها؟)
- Sharjah شارقة
- Dubai دبي
- Ajman عجمان
- Other أخرى

B. ***Questions regarding your knowledge about OTC medications counseling during pregnancy and Breastfeeding:***

What is your recommendation that you will provide for each symptom if being consulted by a pregnant and breastfeeding women?

ما موقفك الطبي في الحالات المرضية التالية التي قد تصيب السيدات خلال فترة الحمل و الرضاعة ؟

	**Condition (الحالة المرضية)**	**Refer to a doctor (إحالة المريض إلى الطبيب)**	**Dispense medicine (صرف دواء)**	**Provide only advice without dispensing medicine (تقديم نصيحة فقط دون صرف أي دواء)**	**Recommending vitamins and food supplements (تقديم نصيحة بصرف فيتامينات و مكملات غذائية)**
	**الحوامل \Pregnant women**				
**8)**	صداع\Headache				
**9)**	cough, runny nose, sore throat سعال ، زكام ، ألم في الحلق				
**10)**	إمساك \Constipation				
**11)**	nausea/vomiting غثيان\إقياء				
**12)**	GERD and indigestion حموضة و عسر هضم				
**13)**	إسهال\Diarrhea				
**14)**	بواسير \Hemorrhoids				
**15)**	أرق \Insomnia				
**16)**	varicose vein الدوالي الوريدية				
**17)**	swelling of the feet and legs انتفاخ في الأرجل و الأقدام				
**18)**	حكة\vaginal itching مهبلية				
**19)**	ألم في \back pain الظهر				
**20)**	حرارة \Fever				

	Condition (الحالة المرضية)	Refer to a doctor (إحالة المريض إلى الطبيب)	Dispense medicine (صرف دواء)	Provide only advice without dispensing medicine (تقديم نصيحة فقط دون صرف أي دواء)	Recommending vitamins and food supplements (تقديم نصيحة بصرف فيتامينات و مكملات غذائية)
	**المرضعات \Breastfeeding Women**				
**21)**	إلتهاب الثدي\Mastitis				
**22)**	sore or cracked تشقق حلمات الثدي\nipple				
**23)**	قلة \insufficient milk إدرار الحليب				
**24)**	صداع\Headache				
**25)**	cough, runny nose, sore throat سعال ، زكام ، ألم في الحلق				
**26)**	إمساك \Constipation				
**27)**	nausea/vomiting غثيان\إقياء				
**28)**	GERD and indigestion حموضة و عسر هضم				
**29)**	إسهال\Diarrhea				
**30)**	بواسير \Hemorrhoids				
**31)**	أرق \Insomnia				
**32)**	varicose vein الدوالي الوريدية				
**33)**	swelling of the feet and legs انتفاخ في الأرجل و الأقدام				
**34)**	حكة\vaginal itching مهبلية				
**35)**	ألم في \back pain الظهر				
**36)**	حرارة \Fever				

C. Questions regarding Services provided regarding self-care in pregnancy and lactation

37) Do you have experience in providing services for pregnant women?

هل لديك خبرة في تقديم الخدمات الطبية للنساء الحوامل؟

- Yes/نعم
- No/لا

(If No experience, please go to question 42/42 إذا لم تكن هناك خبرة ، يرجى الانتقال إلى السؤال)

38) If yes to the previous question, how many pregnant women do receive your services in this pharmacy per week?

إذا كانت الإجابة بنعم على السؤال السابق ، فكم من النساء الحوامل يتلقين خدماتك الطبية في هذه الصيدلية أسبوعيًا؟

- 1
- 2-3
- ≥4

39) If Yes to the previous question, what is the symptom that pregnant women most frequently consulted you about in your pharmacy in the past?

إذا كانت الإجابة "نعم" على السؤال السابق ، فما هو العرض الذي يصيب بشكل متكرر النساء الحوامل في الصيدلية في الماضي؟

- Headache/ back pain/ fever الصداع / آلام الظهر / الحمى
- Constipation/diarrhea الإمساك / الإسهال
- Cough/ runny nose/sore throat السعال / سيلان الأنف / التهاب الحلق
- Nausea/vomiting الغثيان / التقيؤ
- Indigestion/ GERD عسر الهضم / ارتجاع المريء
- Hemorrhoids بواسير
- Other آخر

40) Do you have experience in providing services for breastfeeding women?

هل لديك خبرة في تقديم الخدمات و النصائح الطبية للنساء المرضعات؟

- Yes /نعم
- No/**لا**

(If No experience, please go to section D\إذا لم تكن هناك خبرة ، يرجى الانتقال إلى القسم د)

41) If Yes to the previous question, how many breastfeeding women do receive your services in this pharmacy per week?

إذا كانت الإجابة بنعم على السؤال السابق ، فكم من النساء الحوامل يتلقين خدماتك الطبية في هذه الصيدلية أسبوعيًا؟

- 1
- 2-3
- ≥4

42) If Yes to the previous question, what is the symptom that breastfeeding women most frequently consulted you about in your pharmacy in the past?

إذا كانت الإجابة "نعم" على السؤال السابق ، فما هو العرض الذي يصيب بشكل متكرر النساء المرضعات في الصيدلية في الماضي؟

- Headache/ back pain/ fever الصداع / آلام الظهر / الحمى
- Constipation/diarrhea الإمساك / الإسهال
- Cough/ runny nose/sore throat السعال / سيلان الأنف / التهاب الحلق
- Nausea/vomiting الغثيان / التقيؤ
- Indigestion/ GERD عسر الهضم / ارتجاع المريء
- Insufficient milk قلة إدرار الحليب
- Sore or cracked nipple تشقق حلمات الثدي
- Other آخر

D. *Views about self-care in pregnancy and lactation*

Please choose one option that best describes your own view\ يرجى اختيار خيار واحد يصف أفضل وجهة نظر خاصة بك

		Agree\موافق	Neither disagree or agree\ لا أوافق أو يوافق	Disagree\لا أوافق
43)	**Community pharmacists are qualified to provide advice and an over- the-counter (OTC) therapy to treat common and minor symptoms in pregnant women/** الصيادلة مؤهلون لتقديم المشورة والعلاج دون وصفة طبية (OTC) لعلاج الأعراض الشائعة والثانوية لدى النساء الحوامل			
44)	**Community pharmacists are qualified to provide advice and an OTC therapy to treat common and minor symptoms in breastfeeding women/** الصيادلة مؤهلون لتقديم المشورة والعلاج دون وصفة طبية (OTC) لعلاج الأعراض الشائعة والثانوية لدى النساء المرضعات			
45)	**OTC medicines are safe for pregnancy** الأدوية التي تُباع بلا وصفة طبية هي آمنة لاستخدام الحوامل			
46)	**OTC medicines are safe for breastfeeding** الأدوية التي تُباع بلا وصفة طبية هي آمنة لاستخدام المرضعات			
47)	**I am confident about giving advice and counseling to pregnant women** أنا واثق من تقديم المشورة والنصائح للنساء الحوامل			
48)	**I have sufficient knowledge to solve medication and health problems of pregnant women** لديّ معرفة كافية لحل المشكلات الصحية والداوئية للنساء الحوامل			
49)	**I am confident about giving advice and counseling to breastfeeding women** أنا واثق من تقديم المشورة والنصائح للنساء المرضعات			
50)	**I have sufficient knowledge to solve medication and health problems of breastfeeding women** لديّ معرفة كافية لحل المشكلات الصحية والداوئية للنساء المرضعات			
51)	**Pharmacy school provided appropriate training regarding advice and OTC therapy for pregnant women** قدمت كلية الصيدلة التدريب المناسب فيما يتعلق بالنصائح والعلاج المتاح بدون وصفة طبية للنساء الحوامل			
52)	**Pharmacy school provided appropriate training regarding advice and OTC therapy for breastfeeding women.** قدمت كلية الصيدلة التدريب المناسب فيما يتعلق بالنصائح والعلاج المتاح بدون وصفة طبية للنساء المرضعات			

53) What is /are the most common source(s) of information do you use for responding to symptoms during pregnancy and breastfeeding and /or searching about medicines use in pregnancy and breastfeeding?

ما هو أكثر مصادر المعلومات شيوعًا التي تستخدمها بخصوص الأعراض أثناء الحمل والرضاعة الطبيعية و / أو البحث عن الأدوية التي تستخدمها في الحمل والرضاعة الطبيعية؟

- Books الكتب
- Medical Journal articles مقالات المجلات الطبية
- Websites المواقع الالكترونية
- Others آخر
